# Availability and readiness of healthcare facilities and their effects on antenatal care services uptake in Bangladesh

**DOI:** 10.1186/s12913-024-10824-4

**Published:** 2024-04-05

**Authors:** Md. Nuruzzaman Khan, Md. Badsha Alam, Atika Rahman Chowdhury, Md. Awal Kabir, Md. Mostaured Ali Khan

**Affiliations:** 1 Department of Population Science, Jatiya Kabi Kazi Nazrul Islam University, Namapara, Mymensingh 2220 Bangladesh; 2 School of Medicine and Public Health, College of Health, Medicine and Well-Being, The University of Newcastle, NSW 2308, Callaghan, Australia; 3 Department of Social Work, Pabna University of Science and Technology, Pabna, 6600 Bangladesh; 4 Maternal and Child Health Division (MCHD), icddr,b, 68 Shaheed Tajuddin Ahmed Sarani, Mohakhali, Dhaka, 1212 Bangladesh

**Keywords:** Antenatal care, Healthcare facility level factors, Linked data, Bangladesh

## Abstract

**Background:**

Sustainable Development Goal (SDG) 3.7 aims to ensure universal access to sexual and reproductive healthcare services, where antenatal care (ANC) is a core component. This study aimed to examine the influence of health facility availability and readiness on the uptake of four or more ANC visits in Bangladesh.

**Methods:**

The 2017/18 Bangladesh Demographic and Health Survey data were linked with the 2017 Health Facility Survey and analyzed in this study. The associations of health facility-level factors with the recommended number of ANC uptakes were determined. A multilevel mixed-effect logistic regression model was used to determine the association, adjusting for potential confounders.

**Results:**

Nearly 44% of mothers reported four or more ANC uptakes, with significant variations across several areas in Bangladesh. The average distance of mothers' homes from the nearest health facilities was 6.36 km, higher in Sylhet division (8.25 km) and lower in Dhaka division (4.45 km). The overall uptake of the recommended number of ANC visits was positively associated with higher scores for the management (adjusted odds ratio (aOR) 1.85; 95% CI, 1.16–2.82) and infrastructure (aOR, 1.59; 95% CI, 1.09–2.19) of health facilities closest to mothers' homes. The odds of using the recommended number of ANC in mothers increased by 3.02 (95% CI, 2.01–4.19) and 2.36 (95% CI, 2.09–3.16) folds for each unit increase in the availability and readiness scores to provide ANC services at the closest health facilities, respectively. Every kilometer increase in the average regional-level distance between mothers' homes and the nearest health facilities reduced the likelihood of receiving the recommended number of ANC visits by nearly 42% (aOR, 0.58, 95% CI, 0.42–0.74).

**Conclusion:**

The availability of healthcare facilities close to residence, as well as their improved management, infrastructure, and readiness to provide ANC, plays a crucial role in increasing ANC services uptake. Policies and programs should prioritize increasing the availability, accessibility, and readiness of health facilities to provide ANC services.

**Supplementary Information:**

The online version contains supplementary material available at 10.1186/s12913-024-10824-4.

## Introduction

Maternal mortality is an ongoing public health concern globally, with an estimated 810 deaths every day, mostly from pregnancy-related complications [1]. Over 99% of these deaths occur in Low- and Middle-Income Countries (LMICs), particularly in Southern Asia (66%) and Sub-Saharan Africa (20%), accounting for approximately 86% of the total global occurrence [2]. Almost all of these deaths are preventable if basic and cost-effective maternity care is ensured during pregnancy, delivery, and the postpartum period [3]. As such, ensuring universal access to sexual and reproductive healthcare services, including maternal healthcare services, is an important target to achieve Sustainable Development Goals (SDGs) 3: ensuring health and well-being for all, by 2030.

Antenatal care (ANC) encompasses services provided to pregnant mothers before childbirth and is a crucial maternal healthcare service recommended for all pregnancies. It includes education, screening, counseling, treatment of minor ailments, and immunization services to identify and treat complications through screening and diagnosis. It is also found to be an important determinant of all other types of maternal healthcare services, including delivery and postnatal care [4, 5]. ANC alone is estimated to reduce the maternal mortality rate by up to 20% globally; this contribution is around four times higher (80%) in Low- and Middle-Income Countries (LMICs) [3, 6].

However, despite its importance in ensuring ANC uptake, around 39% of total pregnant women worldwide do not use the recommended number of ANC services [6]. The percentage of non-users is even 1.5 times higher in LMICs (50%), mostly in Asian and African countries [3, 6]. This lower prevalence of ANC in LMICs is also accompanied by poor-quality ANC services [7, 8]. Consequently, dropout from accessing ANC services is very high, and such inadequate care cannot contribute significantly to the reduction of adverse maternal health and birth outcomes, including maternal and under-five mortality [8].

The government of Bangladesh has made universal access to ANC services as a top priority to achieve the health-related SDGs. For this, the government is now provide ANC services in every maternal and child healthcare providing health facilities with either free of costs or with minimal costs [9]. Consequently, ANC services use at least once increased over the years, from 34% in 2000 to 82% in 2017/18, although four or more ANC services uptake is still low at 34% [10]. This lower uptake of four or more ANC services could be due to the poor service quality and need-based use of ANC services only when any complications are faced despite having it’s higher importance to ensure safe pregnancy [11]. The additional challenges are lack of healthcare equipment and personnel, as well as a lower quality of available services [10, 12]. Such challenges have been reported, despite significant progress being achieved during the MDGs, owing primarily to the fixed allocated fund for healthcare services, which causes a mismatch between service availability and demand [13]. Policies and programs are in place to address these challenges in the field level with comprehensive focus of achieving universal healthcare coverage – as outlined in the SDGs goal [14]. Although, such a focus, in general, is making significant progress in healthcare services, however, to what extent such improvement of the healthcare facility affects maternal healthcare services is largely unknown in Bangladesh. A similar limitation has been reported in other LMICs. The underlying reason of such lack is the unavailability of linked healthcare facility and households survey data.

Available research on ANC mainly analysed household level data, collected either by national survey or primary survey, and determined individual-, households-, and community characteristics associated with ANC uptake. These include maternal age and education, partner’s education, number of children ever born, wealth index, access to mass media, place of residence and place of region [15–18]. Healthcare facility-level factors associated with ANC uptake remain largely understudied in Bangladesh, as well as in LMICs. Importantly, such type of analysis of household level data without considering the healthcare facility level factors underestimate or overestimate the true effects of individual-, household- and community level factors on ANC uptake [19, 20]. The underlaying reason is individual-, household-, community-, and healthcare facility level characteristics are interconnected whereas healthcare facility level characteristics work above other factors [20, 21]. To address this limitation, we conducted this study to examine the effects of healthcare facility level factors on ANC uptake in Bangladesh, adjusting for individual-, household-, and community-level factors.

## Methods

### Study setting

In this cross-sectional survey data analysis, we analyzed the 2017/18 Bangladesh Demographic and Health Survey (BDHS) and the 2017 Bangladesh Health Facility Survey (BHFS). Both are nationally representative surveys conducted as part of the Demographic and Health Survey (DHS) program by the United States of America (USA) [22, 23]. The administrative boundary linkage method was used to link the data from these surveys. Through this method, individual respondents were matched and linked with the nearest healthcare facilities situated within the same administrative unit where the respondents resided. In cases where the respondents' homes had the nearest healthcare facilities located in an administrative division other than their own, such selections were excluded. A more comprehensive description of this method has been published elsewhere [22, 23].

The 2017/18 BDHS is the seventh round of DHS in Bangladesh. It was conducted to provide up-to-date information on maternal and child health, including fertility, mortality, and maternal healthcare services use. Nationally representative households were chosen for this survey using stratified random sampling methods in two stages. At the first stage, a total of 675 clusters were selected randomly from a list of 293,579 clusters generated by the Bangladesh Bureau of Statistics as part of the 2011 Bangladesh national population census. Finally, a total of 672 clusters were chosen for the survey, excluding the remaining three clusters due to extreme flooding. The second stage of sampling consisted of selecting a fixed number of 30 households from each of the selected clusters using a simple random sampling method. A list of 20,250 households was generated, of which the survey was conducted in 20,160 households. There were 20,376 eligible women in these selected households with the following conditions: (i) she is a permanent resident of the selected households and passed the most recent night there and (ii) ever married and aged between 15–49 years of age. If a woman spent most of the night at the chosen household but is not a permanent resident of that household, she was also included. With a response rate of 98.8%, a total of 20,127 of the selected women were finally included in the survey.

The 2017 BHFS encompassed 1,524 health facilities, all of which were analyzed in this study. This figure was drawn from a list of 1,600 healthcare facilities generated from the 19,811 registered healthcare facilities in Bangladesh. Each of the 672 PSUs included in the 2017 BDHS and 1,524 healthcare facilities included in the 2017 BHFS has access to GPS point coordinates. Interested readers can see the respective survey reports to know more about these surveys [24, 25].

### Eligible sample

Total of 4,948 mothers who met the inclusion criteria of this study were analysed. The inclusion criteria were: (i) having at least one livebirth within three years prior to the survey and (ii) providing responses to the access of ANC services along with providers of ANC services. All 1,524 healthcare facilities were also included in the analysis.

### Outcome variable

The outcome variable was four or more ANC services use (yes, no). The survey recorded this information by asking women “*Did you receive antenatal healthcare services during your most recent pregnancy?*”. Women who responses positively to this item were then asked, “*How many times did you accessed antenatal healthcare services?*” and “*Where did they receive antenatal healthcare during pregnancy?*”. Women were asked to show the healthcare access card that was used during pregnancy. Number of ANC services received and providers of ANC services, times of accessing ANC services were available in the healthcare access card. If a woman was unable to show the healthcare access card, she was asked several follow-up questions to determine the number, timing, and providers of ANC services. Response recorded were then classified as four or more ANC services access from skilled providers (yes, no).

It is important to mention that we made this classification based on the Bangladesh government’s recommendation of four or more ANC services to receive during pregnancy, rather than the global recommendation of six or more ANC services [26].

### Exposure variables

Health facility-level factors served as a significant exposure variable, with a focus on four key factors: general health service readiness (health facility management systems and infrastructure), degree of availability of antenatal healthcare services at the nearest healthcare facility to mothers' homes, readiness of the nearest healthcare facility to provide antenatal healthcare services, and the average distance between mothers' homes and the nearest healthcare facility (Supplementary Table 1). Continuous scores for each factor were generated based on WHO service availability and readiness indicators [27, 28], and the procedure for creating these scores is detailed elsewhere [29]. The calculation of the distance between mothers' residence clusters and the nearest healthcare facility occurred in two stages. Initially, the distance of each cluster to the nearest healthcare facilities was calculated separately for each of the eight administrative divisions. Subsequently, using road communication data, the average distance between mothers' residence clusters and the nearest healthcare facilities was determined. A comprehensive description of this calculation procedure has been published elsewhere [20, 30].

### Covariates

The covariates considered were selected by reviewing relevant studies on maternal healthcare services access. For this, we first searched several databases, and the variables considered in the identified papers were summarized. The availability of the summarized variables in the survey we analyzed was then checked. Following this, multicollinearity of the available variables was checked. Following these steps, a list of variables was generated where the variables finally chosen were summarized under three headings following the socio-ecological model of health: individual-level factors, household-level factors, and community-level factors [4, 5, 10, 20, 31–34]. Individual-level factors were mother’s age at the birth of the last child, mother’s education status, mother’s employment status, and pregnancy intention at conception. Household-level factors were partner’s education status, partner’s occupation, number of children ever born, intervals between the two most recent live births, and household wealth status. Place of residence and region (administrative division) were considered in community-level factors.

### Statistical analysis

Descriptive statistics were employed to characterize the respondents' features. The association between exposure and outcome variables was assessed using a multilevel mixed-effect logistic regression model at three levels (individuals, households, and clusters), adjusting for factors at the individual, household, and community levels. The rationale for employing the multilevel mixed-effect binary logistic regression model was the hierarchical structure of the BDHS data, where individuals are nested within a household, and households are nested within a cluster [35]. Ignoring this hierarchy in the model could result in less precise estimates. We ran a total of four models. The initial model, referred to as the null model, included only the outcome variable: the utilization of four or more ANC services. In the second model, healthcare facility-level factors were introduced as independent variables, while the utilization of four or more ANC services remained the dependent variable. This association was then expanded upon in the third and fourth models, where individual and household, as well as community-level factors, respectively, were incorporated as additional independent variables. We also conducted a stratified analysis to assess the association of four or more ANC uptakes with healthcare facility-level factors. Stratification was performed based on the place of residence and the average distance between DHS clusters to the nearest ANC services providing healthcare facility in Bangladesh. Results are reported as odds ratios (OR) with 95% confidence intervals (95% CI). All analyses were performed using the statistical package R (version 4.10).

## Results

### Background characteristics

Background characteristics of the respondents are presented in Table 1. Nearly 44% of all respondents reported using four or more ANC services. The rate was even higher among mothers aged 20 to 34 (44.9%, 95% CI, 42.5–47.33) and among unemployed mothers (45.69%, 95% CI, 43.04–48.40). Mothers who had a desired pregnancy at conception reported a higher rate of ANC service utilisation (46.53%, 95% CI, 44.23–48.84) followed by mothers who had a mistimed (40.34%, 95% CI, 35.60–36.51) or an unwanted (27.60, 95% CI, 22.90–32.84) pregnancy at conception. The prevalence of ANC services uptake was found to be much higher among mothers whose partner had more than secondary level education (70.61%, 95% CI, 66.71–74.23) and who were service holders (70.8%, 95% CI, 64.50–73.66). We found lower ANC uptake in mothers who have more than 2 ever born children (33.50%, 95% CI, 30.50–36.70) in comparison to those who had fewer children 48.60% (46.20–51.00). Of the mothers of richest wealth quintile, over 71% (95% CI, 67.32–74.41) reported they uptake four more ANC services. Higher prevalence of four or more ANC services uptake was also reported for urban mothers (56.60%, 95% CI, 52.40–60.70) and mothers whose region of residence were either Khulna (55.23, 95% CI, 49.24—61.07) or Rangpur (52.06%, 95% CI, 56.15–57.91) (Table 1).

**Table 1:** Background characteristics of the respondents, BDHS 2017/18

Characteristics	Overall % (95% CI)^a^	Four or more ANC use, % (95% CI)^b^
**Women’s age at birth of the last child**		
≤19 years	25.14 (23.9-26.6)	43.80 (40.13-47.5)
20-34 years	70.63 (3.67-4.86)	44.90 (42.5-47.33)
≥35 years	4.23 (3.67-4.86)	35.52 (28.62-43.10)
**Women’s education status**		
No formal education	6.33 (5.49-7.28)	16.31 (12.4-21.23)
Primary	27.7 (25.9-29.6)	29.4 (26.3-32.7)
Secondary	48.83 (47-50.7)	47.98 (45.4-50.63)
Higher	17.20 (15.7-18.8)	44.21 (42.02-46.43)
**Women’s employment status **		
Yes	37.20 (35.06-39.4)	41.72 (38.83-44.7)
No	62.8 (60.6-64.94)	45.69 (43.04-48.4)
**Pregnancy intention at conception **		
Wanted	79.09 (77.81-80.32)	46.53 (44.23-48.84)
Mistimed	12.91 (11.91-14.00)	40.34 (35.60-45.3)
Unwanted	8.00 (7.16-8.92)	27.60 (22.9-32.84)
**Partner’s education status**		
No formal education	13.7 (12.3-15.2)	26.5 (22.42-30.91)
Primary	33.61(31.9-35.4)	33.50 (30.60-36.51)
Secondary	34.00 (32.4-35.7)	48.22 (45.20-51.30)
Higher	18.8 (17.00-20.54)	70.61 (66.71-74.23)
**Partner’s occupation**		
Agriculture worker	19.44 (17.81-21.17)	31.90 (28.4-35.6)
Physical worker	53.08 (51.2-55)	43.10 (40.41-45.72)
Services	5.81 (5.07-6.65)	70.80 (64.5-76.33)
Business	20.65 (19.2-22.2)	52.41 (48.33-56.5)
Other	0.22 (0.11-0.43)	56.90 (23.81-84.8)
**Number of children ever born**		
1-2 children	71.02 (6.94-72.6)	48.60 (46.2-51)
>2 children	28.98 (27.41-30.6)	33.50 (30.5-36.7)
**Intervals between the two most recent live births**		
≤2 years	6.80 (6.02-7.7)	34.51 (28.8-40.74)
3-4 years	17.80 (16.6-19.04)	34.90 (28.8-40.74)
>4 years	75.42 (74.00-76.83)	34.90 (31.13-38.84)
**Household wealth status **		
Poorest	20.63 (18.6-22.83)	26.14 (23.03-2951)
Poorer	20.60 (19.04-22.15)	32.13 (28.73-35.73)
Middle	19.18 (17.7-20.8)	42.95 (39.33-46.7)
Richer	20.14 (18.4-22.01)	50.33 (46.4-54.3)
Richest	19.50 (17.6-21.60)	71.00 (67.32-74.41)
**Place of residence**		
Urban	26.80 (25.1-28.52)	56.60 (52.40-60.70)
Rural	73.22 (71.5-74.9)	39.70 (37.15-42.3)
**Region (administrative division)**		
Barishal	5.70 (5.14 -6.32)	36.43 (30.33-43.01)
Chattogram	21.22 (19.6-23.00)	36.74 (31.9-41.9)
Dhaka	25.72 (24.00-27.54)	49.40 (44.3-54.5)
Khulna	9.18 (8.32-10.12)	55.23 (49.24-61.07)
Mymensingh	8.60 (7.7-9.5)	38.63 (33.35-44.18)
Rajshahi	11.53 (10.3-12.9)	45.90 (39.4-52.54)
Rangpur	10.53 (9.5-11.7)	52.06 (46.15-57.91)
Sylhet	7.55 (6.69-8.51)	32.9 (26.24-40.3)

^a^Column percentage

^b^Row percentage

### Distribution of health facilities in Bangladesh

Table 2 presents the distribution of health facilities across divisions. Out of the 1524 health facilities, 1413 (92.71%) offered ANC services. The average distance between these health facilities and the BDHS clusters was approximately 6.36 km, with the highest in the Sylhet division (8.25 km) and the lowest in the Dhaka division (4.45 km). The health facility management system's mean score was 0.81, with the Dhaka division (0.94) scoring higher than Khulna (0.89), Rangpur (0.85), and Mymensingh (0.85) divisions. Similarly, Dhaka (mean score, 0.87) and Rangpur (mean score, 0.72) reported higher availability of various antenatal healthcare services compared to the overall average score of 0.70. The average score for healthcare facility readiness to provide ANC services was 0.67, with the lowest reported in Sylhet (0.46) and Barishal (0.51) divisions.

**Table 2:** Division-wise distribution of health facilities, antenatal healthcare services availability, readiness to provide antenatal healthcare services, and health average distance from the demographic and health survey programme clusters in Bangladesh

**Division**	**Availability of antenatal healthcare services (*N*=1524)**		**Average distance between home and health facility (km)**	**Health facility management**	**Health facility infrastructure**	**Severity of the availability of antenatal healthcare services at the nearest health care facility**	**Health care facility readiness to provide antenatal healthcare services**
	**Yes (*n*=1413)**	**No (*n*=111)**					
Barishal	117 (99.15)	1 (0.85)	6.46	0.67	0.80	0.68	0.51
Chattogram	266 (93.99)	17 (6.01)	5.92	0.78	0.86	0.71	0.83
Dhaka	280 (93.33)	20 (6.67)	4.45	0.94	0.94	0.87	0.87
Khulna	183 (94.82)	10 (5.18)	5.9	0.89	0.9	0.68	0.58
Rajshahi	205 (91.91)	11 (5.09)	7.28	0.80	0.79	0.61	0.7
Rangpur	150 (79.79)	40 (20.21)	5.9	0.85	0.91	0.72	0.85
Sylhet	100 (96.15)	4 (3.85)	8.25	0.68	0.68	0.66	0.46
Mymensingh	112 (91.80)	10 (8.20)	6.71	0.85	0.77	0.64	0.57
**Grand average distance **			**6.36**	**0.81**	**0.83**	**0.70**	**0.67**

### Model selection

The results of the multilevel mixed-effect logistic regression model assessing the association of ANC services uptake with health facility, individual, household, and community-level factors are presented in Table 3. Four separate models were run, and the best model is the one with the smallest AIC, BIC, and ICC values. Based on these markers, the final model, model 4, was the best-fitted model. The null model's ICC value was 0.16213, indicating a 16.21% difference in four or more ANC uptakes across clusters considered in the analysis. This reduced to only 6.87% once health facility, individual, family, and community-level factors were adjusted in the final model. The variance of the random intercept decreased from 2.02 to 1.26 as we progressed from the null model to model 4. This decline further indicates that model 4 provided a better fit to the data compared to the previous models.

**Table 3:** Multi-level logistic regression model with health facility level factors as the sole correlates of four or more antenatal care services use

**Characteristics**	**Null model**	**Health facility-level model, aOR (95% CI)**	**Health facility-, individual-, and household-level model, aOR (95% CI)**	**Health facility-, individual-, household-, and community-level model, aOR (95% CI)**
**General health service readiness**				
Health facility management system		1.96 (1.10-3.16)^**^	1.90 (1.14-3.01)^**^	1.85 (1.16-2.82)^**^
Health facility infrastructure		2.12 (1.36-3.40)^**^	1.86 (1.19-2.26)^***^	1.59 (1.09-2.19)^**^
**Degree of availability of antenatal healthcare services at the nearest healthcare facility to mothers' homes**		3.96 (2.46-5.68)^***^	3.18 (2.18-5.19)^**^	3.02 (2.01-4.19)^**^
**Readiness of the mothers’ homes nearest healthcare facility to provide antenatal healthcare services**		4.16 (2.86-4.98)^**^	3.16 (2.18-3.72)^***^	2.36 (2.09-3.16)^**^
**Average distance between mothers' homes and the nearest healthcare facility**		0.54 (0.40-0.82)^***^	0.57 (0.43-0.78)^***^	0.58 (0.42-0.74)^***^
**Women’s age in years**				
≤19 (ref)			1.00	1.00
20-34			1.01( 0.83-1.24)	1.00 ( 0.82-1.22)
≥35			0.90 (0.55-1.39)	0.87 ( 0.54-1.40)
**Women’ education status**				
No formal education (Ref)			1.00	1.00
Primary			1.66 (1.10-2.48)^**^	1.74 ( 1.16-2.60)^**^
Secondary			2.25 (1.49-3.39)^**^	2.32 (1.54-3.50)^**^
Higher			2.36 (1.48-3.76)^***^	2.49 (1.56-3.98)^**^
**Women’ employment status**				
No (ref)			1.00	1.00
Yes			1.43 (1.19-1.72)^**^	1.24 (1.03-1.50)^**^
**Pregnancy intention at conception**				
Wanted (Ref)			1	
Mistimed			0.94 (0.74-1.19)	0.85 (0.67-1.08)
Unwanted			0.72 (0.52-0.99)^***^	0.66 (0.47-0.91)^**^
**Quality of Antenatal care **				0.33 (0.30-0.37)^***^
**Husbands’ education status**				
No education (ref)			1.00	1.00
Primary			0.97 (0.72-1.31)	0.97 (0.72-1.31)
Secondary			1.02 (0.74-1.39)	1.06 (0.78-1.45)
Higher			1.61 (1.10-2.34)^***^	1.53 (1.05-2.22)^**^
**Husbands’ occupation**				
Agricultural worker (ref)			1.00	1.00
Physical worker			3.04 (1.38-6.69)^**^	2.98 (1.27-6.98)^**^
Services			3.02 (1.28-7.16)^**^	2.90 (1.16-7.45)^**^
Business			3.25 (1.46-7.25)^**^	3.17 (1.33-7.56)^**^
Other			2.76 (1.27-6.15)^**^	2.15 (1.18-4.25)^***^
**Number of children ever born**				
1-2 children(ref)			1	1
>2 children			1.08 (0.85-1.35)	1.17 (0.93-1.47)
**Intervals between the two most recent live births **				
≤2 years			1.00	1.00
3-4 years			1.04 (0.72-1.50)	1.06 (0.73-1.54)
>4 years			1.34 (0.94-1.91)	1.21 (0.85-1.73)
**Household wealth status**				
Poorest (ref)			1	1.00
Poorer			0.99 (0.76 ,1.29)	1.03 (0.78-1.35)
Middle			1.14 (0.89-1.46)	1.25 (0.97-1.62)
Richest			1.87 (1.38-2.55)	2.06 (1.48-2.85)^**^
Richer			1.14 (0.88-1.48)	1.27 (0.97-1.66)
**Place of residence**				
Urban (ref)				1
Rural				0.82 (0.67-1.00)
**Region (administrative division)**				
Barisal (ref)				1
Chattogram				0.77 (0.54-1.08)
Dhaka				1.21 (0.85-1.71)
Khulna				1.77 (1.23-2.54)^***^
Mymensingh				1.48 (1.01-2.20)^**^
Rajshahi				1.49 (1.02-2.17)^**^
Rangpur				2.90 (1.98-4.25)^**^
Sylhet				1.02 (0.67-1.58)
**Model summary**				
AIC	1832.36	1623.42	1512.38	1428.12
BIC	1642.12	1414.15	1335.47	1236.56
ICC	16.21%	12.25%	9.46%	6.87%
Median Odds Ratio	2.12	2.02	1.98	1.68
Variance of the random intercept	2.02 (0.36)	1.96 (0.43)	1.46 (0.32)	1.26 (0.25)

***p<0.001, **p<0.01

### Association of four or more antenatal care service uptake with health facility-, individual-, family- and community-level factors

The association between the uptake of four or more ANC services and healthcare facility-, individual-, family-, and community-level factors determined through four models is presented in Table 3. However, since model 4 demonstrated the best fit, we summarize these results here, noting that the findings in other models were directionally similar. We observed that each unit increase in the management and infrastructure scores of the nearest healthcare facility was associated with 1.85 times (95% CI, 1.16–2.82) and 1.59 times (95% CI, 1.09–2.19) higher odds/likelihood of adequate ANC uptake, respectively. The likelihood of ANC service access increased (aOR, 3.02, 95% CI, 2.01—4.19) with an increase in the availability of ANC services at the healthcare facility nearest to mothers’ homes. Similarly, each unit increase in the score of readiness of mothers’ homes nearest healthcare facility was associated with 2.36 times (95% CI, 2.09–3.16) increase in the likelihood of four or more ANC services uptake. Furthermore, we found that for every one-kilometre increase in distance between mothers' homes and the nearest healthcare facility, there was a 42% decrease in the uptake of four or more ANC services.

Of the different covariates adjusted in the final model, mothers' increased years of education, their partner’s occupation other than agricultural workers, a richer wealth quintile, and residing in Khulna, Mymensingh, Rajshahi, and Rangpur were found to be positively associated with an increased uptake of four or more ANC services. In contrast, lower quality of ANC services and experiencing mistimed and unwanted pregnancies rather than wanted pregnancies were found as negative predictors for the uptake of four or more ANC services.

### Health facility environment and uptake of four or more antenatal healthcare services

We generated the health facility environment by considering the number of ANC services providing healthcare facilities within 6.36 km (the average distance between DHS cluster to the nearest ANC services providing healthcare facility in Bangladesh). Following this, we ran three different multilevel mixed-effects logistic regression models for overall, rural, and urban areas to access the relationships between four or more ANC service uptake and the health facility environment. Individual, household, and community-level factors were adjusted in each model (Table 4). We found the likelihood of ANC services uptake increased with the increased number of healthcare facilities within 6.36 km, and the relationship reported was strongest for the rural area. Increased healthcare facility scores, in response to the management and infrastructure, were found as significant influential predictors of ANC services uptake for all cases, though effect sizes were strongest for rural areas following urban areas and overall. Similarly, the availability of ANC services and the readiness of mothers’ homes nearest healthcare facility to provide ANC services were reported as significant predictors of ANC services uptake for overall and for rural and urban areas separately. However, as before, the relationship was strongest for rural areas.

**Table 4:** Multilevel logistics regression assessing the relationships between of four or more antenatal care services use and attributes of health facilities located within 6.36 km distance from the BDHS clusters

**Health facility characteristics **	**Four or more Antenatal care services use, aOR (95% CI)**		
	**Overall**^**a**^	**Rural**^**a**^	**Urban**^**a**^
**Number of health facilities**			
0	1.00	1.00	---
1	2.26 (2.04-3.12)^***^	3.12 (2.48-5.12)^**^	1.00
≥2	3.12 (1.89-5.14)^**^	4.54 (2.54-5.68)^**^	2.26 (1.95-3.98)^**^
**General health service readiness**			
Health facility management system	2.54 (1.17-4.18)^**^	3.17 (1.98-4.56)^**^	1.19 (0.95-2.98)^**^
Health facility infrastructure	2.75 (1.47-3.78)^**^	3.75 (1.50-4.98)^**^	2.15 (1.46-4.15)^**^
**Degree of availability of antenatal healthcare services at the nearest healthcare facility to mothers' homes**	4.12 (2.01-6.15)^***^	5.18 (2.15-6.98)^***^	3.18 (1.18-5.78)^**^
**Readiness of the mothers’ homes nearest healthcare facility to provide antenatal healthcare services**	3.98 (2.14-5.50)^**^	5.98 (3.19-6.52)^**^	2.17 (1.78-4.56)^**^

^a^Models adjusted with women’s age, education, employment status, number of children ever born, intervals between the two most recent live births, husbands’ education, occupation, pregnancy intention at conception, household wealth quintile, place of residence and region

***p<0.001, **p<0.01

## Discussion

This study found that improving healthcare facility management and infrastructure, along with increased availability of ANC services at the healthcare facility nearest to mothers' homes and its readiness to provide ANC services, are significant determinants of the uptake of four or more ANC services. We also found that every kilometre increase in distance between mothers' homes and the nearest healthcare facility where ANC services are available is associated with a 42% lower odds of uptake for four or more ANC services. These relationships were observed for Bangladesh as a whole, as well as for its urban and rural areas separately, with the rural areas exhibiting the strongest association. The findings will assist policymakers in prioritizing improvements in healthcare facilities to achieve the SDGs' goal of universal healthcare coverage and a reduction in maternal and child mortality by 2030.

The study found that 44% of women used ANC four or more times, which is 12% higher than the prevalence reported in 2014 [33], and consistent with prior evidence, socio-economic variation was observed as well [33, 34, 36–39]. Although the increasing trend indicates progress in ANC uptake, the growth rate is insufficient to meet the SDGs targets by 2030—merely 6 years from now. Moreover, the rate still remains lower than in neighboring countries such as India (51.7%), Nepal (69.8%), and Pakistan (36%) [40–42]. Additional context-specific policies and programs are now a priority for Bangladesh to increase ANC services uptake to meet the SDGs target by 2030.

We revealed that improving healthcare facility-level factors, including better management and infrastructure, availability of ANC services at the nearest healthcare facility, and its readiness to provide ANC services, play a significant role in increasing the uptake of ANC services. The findings could not be directly validated due to the lack of relevant literature in Bangladesh and other LMICs. However, previous studies in Bangladesh reported a significant influence of healthcare facility-level factors on modern contraception uptake, reducing cesarean delivery, unintended pregnancy, and improving pregnancy outcomes [19, 20, 43–46]. The association may arise in both direct and indirect ways. Existing evidence showed that adequate infrastructure and the availability of healthcare providers and equipment are key indicators of quality care [12], which later increases ANC services uptake [47–49]. In line with our findings, this pathway is commonly observed in Bangladesh and LMICs [50, 51].

In Bangladesh, current governmental initiatives to enhance healthcare facility management and infrastructure, as well as the increasing number of healthcare facilities, are on their way to increasing ANC service uptake, although there are challenges that need to be addressed to get best effectiveness of the healthcare facility. For example, a common challenge in Bangladesh is the lack of skilled and enough healthcare personnel. This low provider-patient ratio frequently increases waiting time in facilities, which becomes a burden for pregnant women during ANC check-ups. These circumstances altogether contribute to women dropping out of ANC services. Another significant barrier to ANC service uptake is healthcare facility readiness to provide ANC, which frequently arises as a result of lower governmental priorities to equip Upazila to community level healthcare facilities with required facilities. According to a recent study in Bangladesh, 54.6% of facilities that provide ANC services have at least one ANC-trained staff, while only 4.3% of facilities are fully prepared to provide ANC services [31]. Although, in total around 13,000 community clinics are available across rural areas of the country and only 33% of the facilities have considerably high preparedness for ANC services [31]. By policy, they are the primary provider of ANC services, but their lack of readiness impedes women's access to healthcare. All of these factors have a negative impact on women's ANC uptake behaviours, as reflected in our analysis and consistent with other relevant study findings for other outcomes in Bangladesh [19, 20, 43].

However, we found that the increase in distance of mothers’ homes to the nearest healthcare facility reduces women’s ANC services uptake, especially in the rural areas. Our findings corroborates with the findings from other LMICs settings [33, 34, 36–39, 52–56]. There are several possible explanations for this influence. For instance, increased travel time and costs may contribute to the uptake of healthcare services, particularly for ANC services, which require multiple visits. Moreover, mothers in Bangladesh, like in LMICs, frequently face mobility restrictions due to community-level misconceptions and societal norms [19, 20]. This is particularly more evident in the late stages of pregnancy. In addition, there is a tendency in Bangladesh, like in many other LMICs, to access healthcare services based on need rather than considering merits [44]. This, together with the increased distance, can then contribute to lower uptake of ANC services until serious complications arise. These challenges are common countrywide, particularly in rural areas and among mothers facing socio-economic disadvantages [19, 20, 44].

This study holds significant policy implications aimed at enhancing the utilization of antenatal healthcare services and improving maternal healthcare outcomes. The notable correlation between healthcare facility-level factors and ANC uptake underscores the importance of prioritizing healthcare services alongside the current focus on individual, household, and community-level factors. This necessitates governmental attention towards healthcare services, including enhancing service availability and readiness of healthcare facilities through proper monitoring and strengthening existing infrastructure. The findings also highlight the potential impact of reducing the distance to healthcare facilities on increasing healthcare service uptake. This underscores the importance of initiating ANC services in community clinics across Bangladesh, which serve as primary healthcare centres catering to approximately 6000 individuals. However, to achieve this, the government must ensure the presence of healthcare personnel at these facilities, coupled with infrastructural development efforts. Collectively, these initiatives are poised to bolster ANC uptake towards universal levels, thereby reducing maternal and child mortality rates and advancing the relevant SDGs.

### Strengths and limitations

This study has several strengths and a few limitations. First and foremost, as a strength, the study examined two nationally representative datasets that are relatively large and representative of all areas. Second, we used an advanced statistical model to analyze the data, adjusting for potential confounders, which has made the study findings more precise and reliable. However, the major limitation is that the cross-sectional nature of the BDHS survey restricted the study from establishing a causal relationship between exposures and outcomes. The BDHS displaced cluster locations up to 0–5 km for rural areas and 0–2 km for urban areas to ensure the privacy of the respondents. As a result, the calculated average distance between the nearest health facility and the actual distance may differ slightly. The BDHS, on the other hand, ensured that the new disrupted locations remained within the designated administrative boundaries. As a result, errors due to displacement are likely to be random and small. A previous study found that this variation has no effect [57]. Recall bias is another issue that might have occurred during reported ANC visits and other confounding factors. However, despite these limitations, this is the first study in the context of Bangladesh and other LMICs that explored the effects of health facility-level factors on ANC services uptake in Bangladesh, adjusted for individual, household, and community-level factors. This will help policymakers in Bangladesh and other LMICs to understand why prioritizing healthcare facilities is important to ensure the recommended number of ANC services uptake.

## Conclusion

This study revealed a lower uptake of the recommended four or more ANC services. Factors at the healthcare facility level, such as the availability of ANC services, the readiness of healthcare facilities to provide these services, and the proximity of mothers' homes to the nearest healthcare facility, emerged as crucial determinants of the uptake of four or more ANC services. This suggests that significant investment in the healthcare sector is required to ensure the availability of maternal healthcare services at the root level healthcare facilities, such as community clinics. Adequate skilled healthcare personnel need to be recruited and ensured to be present at the community healthcare facility on a 24/7 basis. Health facilities should be strengthened to provide ANC services, and more health facilities with such capacities are needed. For this, policies and programs should prioritize increasing the availability and accessibility of health facilities that provide ANC services.

### Supplementary Information

**Supplementary Material 1.**

## Data Availability

The data that support the findings of this study are available from The DHS Program, but restrictions apply to the availability of these data, which were used under license for the current study, and so are not publicly available. Data are, however, available from the authors upon reasonable request and with permission of The DHS Program (https://dhsprogram.com/data/). Data are, however, available from the corresponding author (Khan MMA via email: mostaured.khan@gmail.com) upon reasonable request and with permission of the DHS Program.

## Abbreviations

LMICs: Low- and middle-income countries
SDG: Sustainable development goal
ANC: Antenatal care service
BDHS: Bangladesh demographic and health survey
BHFS: Bangladesh health facility survey
OR: Odds ratio
CI: Confidence interval
AIC: Akaike information criterion
BIC: Bayesian information criterion

## References

[1] Ahinkorah BO, Seidu A-A, Budu E, Mohammed A, Adu C, Agbaglo E, Ameyaw EK, Yaya S (2022). Factors associated with the number and timing of antenatal care visits among married women in Cameroon: evidence from the 2018 Cameroon Demographic and Health Survey. J Biosoc Sci.

[2] Organization WH (2019). Trends in maternal mortality 2000 to 2017: estimates by WHO.

[3] World Health Organization (2022). Maternal Mortality.

[4] Khan MN, Harris ML, Loxton D (2020). Assessing the effect of pregnancy intention at conception on the continuum of care in maternal healthcare services use in Bangladesh: Evidence from a nationally representative cross-sectional survey. PLoS ONE.

[5] Khan MN, Harris ML, Loxton D (2022). Low utilisation of postnatal care among women with unwanted pregnancy: A challenge for Bangladesh to achieve Sustainable Development Goal targets to reduce maternal and newborn deaths. Health Soc Care Community.

[6] Hossain AT, Siddique AB, Jabeen S, Khan S, Haider MM, Ameen S, Tahsina T, Chakraborty N, Nahar Q, Jamil K (2023). Maternal mortality in Bangladesh: Who, when, why, and where? A national survey-based analysis. J Global Health.

[7] Kuhnt J, Vollmer S (2017). Antenatal care services and its implications for vital and health outcomes of children: evidence from 193 surveys in 69 low-income and middle-income countries. BMJ Open.

[8] Merid MW, Chilot D, Yigzaw ZA, Melesse AW, Ferede MG, Aragaw FM, Bitew DA (2024). Women in low-and middle-income countries receive antenatal care at health institutions, yet not delivered there: a multilevel analysis of 2016–2021 DHS data. Trop Med Health.

[9] Khan MN, Harris M, Loxton D (2020). Modern Contraceptive Use Following an Unplanned Birth in Bangladesh: An Analysis of National Survey Data. Int Perspect Sex Reprod Health.

[10] Mn KHAN (2020). Effects of Unintended Pregnancy on Maternal Healthcare Services Use in Bangladesh.

[11] Sarker BK, Rahman M, Rahman T, Rahman T, Khalil JJ, Hasan M, Rahman F, Ahmed A, Mitra DK, Mridha MK (2020). Status of the WHO recommended timing and frequency of antenatal care visits in Northern Bangladesh. PLoS ONE.

[12] Kruk ME, Gage AD, Arsenault C, Jordan K, Leslie HH, Roder-DeWan S, Adeyi O, Barker P, Daelmans B, Doubova SV (2018). High-quality health systems in the Sustainable Development Goals era: time for a revolution. Lancet Glob Health.

[13] Beattie R, Brown N, Cass H (2015). Millennium development goals progress report. Arch Dis Child.

[14] Joarder T, Chaudhury TZ, Mannan I. Universal health coverage in Bangladesh: activities, challenges, and suggestions. Adv Public Health. 2019;1–12. 10.1155/2019/4954095 PMC7691757 33281233

[15] Rahman A, Nisha MK, Begum T, Ahmed S, Alam N, Anwar I (2017). Trends, determinants and inequities of 4+ ANC utilisation in Bangladesh. J Health, Popul Nutr.

[16] Sarker BK, Rahman T, Rahman T, Rahman M (2021). Factors associated with the timely initiation of antenatal care: findings from a cross-sectional study in Northern Bangladesh. BMJ Open.

[17] Ali N, Sultana M, Sheikh N, Akram R, Mahumud RA, Asaduzzaman M, Sarker AR (2018). Predictors of optimal antenatal care service utilization among adolescents and adult women in Bangladesh. Health Serv Rep and Managerial Epidemiol.

[18] Pervin J, Venkateswaran M, Nu UT, Rahman M, O’Donnell BF, Friberg IK, Rahman A, Frøen JF (2021). Determinants of utilization of antenatal and delivery care at the community level in rural Bangladesh. PLoS ONE.

[19] Khan N, Islam Trisha N, Rashid M. Availability and readiness of health care facilities and their effects on under-five mortality in Bangladesh: Analysis of linked data. J Glob Health. 2022;12:04081. 10.7189/jogh.12.04081 PMC9480612 36112406

[20] Khan MN, Akter S, Islam MM. Availability and readiness of healthcare facilities and their effects on long-acting modern contraceptive use in Bangladesh: analysis of linked data. BMC Health Serv Res. 2022;22(1):1180. 10.1186/s12913-022-08565-3 PMC9490900 36131314

[21] Kilanowski JF (2017). Breadth of the socio-ecological model. J Agromedicine.

[22] Burgert  CR, Prosnitz Debra (2014). Linking DHS Household and SPA Facility Surveys: Data Considerations and Geospatial Methods. DHS Spatial Analysis Reports No. 10.

[23] Kanyangarara M, Chou VB, Creanga AA, Walker N (2018). Linking household and health facility surveys to assess obstetric service availability, readiness and coverage: evidence from 17 low-and middle-income countries. J Global Health..

[24] National Institute of Population Research and Training (NIPORT) and ICF (2017). Bangladesh Health Facility Survey.

[25] National Institute of Population Research and Training (NIPORT) and ICF. (2017). Bangladesh Demography and Health Survey.

[26] Directorate General of Health Services (DGHS) (2019). Bangladesh national strategy for maternal health 2019–2030. Dhaka, Bangladesh Ministry of Health and Family Welfare.

[27] Organization WH (2013). Service availability and readiness assessment (SARA): an annual monitoring system for service delivery: reference manual.

[28] O'Neill K, Takane M, Sheffel A, Abou-Zahr C, Boerma T (2013). Monitoring service delivery for universal health coverage: the Service Availability and Readiness Assessment. Bull World Health Organ.

[29] Cummins D (2017). Teenage pregnancy and early marriage in Timor-Leste: Research on the decision-making pathways of young women in the Municipalities of Covalima.

[30] Tegegne TK, Chojenta C, Forder PM, Getachew T, Smith R, Loxton D (2020). Spatial variations and associated factors of modern contraceptive use in Ethiopia: a spatial and multilevel analysis. BMJ Open.

[31] Hakim S, Chowdhury MAB, Ahmed Z, Uddin MJ (2022). Are Bangladeshi healthcare facilities prepared to provide antenatal care services? Evidence from two nationally representative surveys. PLOS Glob Public Health.

[32] Khan MA, Khan N, Rahman O, Mustagir G, Hossain K, Islam R, Khan HT (2021). Trends and projections of under-5 mortality in Bangladesh including the effects of maternal high-risk fertility behaviours and use of healthcare services. PLoS ONE.

[33] Khan MN, Harris ML, Oldmeadow C, Loxton D (2020). Effect of unintended pregnancy on skilled antenatal care uptake in Bangladesh: analysis of national survey data. Arch Public Health.

[34] Talukder A, Siddiquee T, Noshin N, Afroz M, Ahammed B, Halder HR (2021). Utilization of Antenatal Care Services in Bangladesh: A Cross-sectional Study Exploring the Associated Factors. The Anatolian J FamMed.

[35] Diez-Roux AV (2000). Multilevel Analysis in Public Health Research. Annu Rev Public Health.

[36] Chanda SK, Ahammed B, Howlader MH, Ashikuzzaman M (2020). Shovo T-E-A, Hossain MT: Factors associating different antenatal care contacts of women: A cross-sectional analysis of Bangladesh demographic and health survey 2014 data. PLoS ONE.

[37] Islam MM, Masud MS (2018). Determinants of frequency and contents of antenatal care visits in Bangladesh: Assessing the extent of compliance with the WHO recommendations. PLoS ONE.

[38] Islam MA, Kabir MR, Talukder A (2020). Triggering factors associated with the utilization of antenatal care visits in Bangladesh: An application of negative binomial regression model. Clin Epidemiol Glob Health.

[39] Bhowmik KR, Das S, Islam MA (2020). Modelling the number of antenatal care visits in Bangladesh to determine the risk factors for reduced antenatal care attendance. PLoS ONE.

[40] Ogbo FA, Dhami MV, Ude EM, Senanayake P, Osuagwu UL, Awosemo AO, Ogeleka P, Akombi BJ, Ezeh OK, Agho KE (2019). Enablers and barriers to the utilization of antenatal care services in India. Int J Environ Res Public Health.

[41] Neupane B, Rijal S, Gc S, Basnet TB (2020). Andersen’s model on determining the factors associated with antenatal care services in Nepal: an evidence-based analysis of Nepal demographic and health survey 2016. BMC Pregnancy Childbirth.

[42] Sahito A, Fatmi Z (2018). Inequities in antenatal care, and individual and environmental determinants of utilization at national and sub-national level in Pakistan: a multilevel analysis. International J Health Policy Manag.

[43] Khan M, Harris ML, Huda M, Loxton D (2022). A population-level data linkage study to explore the association between health facility level factors and unintended pregnancy in Bangladesh. Sci Rep.

[44] Khan MN, Islam MM, Akter S (2023). Spatial distribution of caesarean deliveries and their determinants in Bangladesh evidence from linked data of population and health facility survey. Lancet Reg Health Southeast Asia..

[45] Kabir MA, Rahman MM, Khan MN (2022). Maternal anemia and risk of adverse maternal health and birth outcomes in Bangladesh: A nationwide population-based survey. PLoS ONE.

[46] Khan MN, Kabir MA, Shariff AA, Rahman MM (2022). Too many yet too few caesarean section deliveries in Bangladesh: Evidence from Bangladesh Demographic and Health Surveys data. PLOS Global Public Health.

[47] Zhongming Z, Linong L, Xiaona Y, Wangqiang Z, Wei L (2018). Low quality healthcare is increasing the burden of illness and health costs globally.

[48] Tadesse Berehe T, Modibia LM (2020). Assessment of quality of antenatal care services and its determinant factors in public health facilities of Hossana town.

[49] Puspitasari DA, Omas Bulan S (2021). The effect of accessibility and availability of health infrastructure on maternal healthcare utilization in Indonesia to achieve Sustainable Development Goals. IOP Conference Series: Earth Environ Sci.

[50] Negash WD, Fetene SM, Shewarega ES, Fentie EA, Asmamaw DB, Teklu RE, Aragaw FM, Belay DG, Alemu TG, Eshetu HB (2022). Multilevel analysis of quality of antenatal care and associated factors among pregnant women in Ethiopia: a community based cross-sectional study. BMJ Open.

[51] Anwar I, Nababan HY, Mostari S, Rahman A, Khan JA (2015). Trends and inequities in use of maternal health care services in Bangladesh, 1991–2011. PLoS ONE.

[52] Fisseha G, Berhane Y, Worku A, Terefe W (2017). Distance from health facility and mothers’ perception of quality related to skilled delivery service utilization in northern Ethiopia. Int J Women's Health.

[53] Dotse-Gborgbortsi W, Dwomoh D, Alegana V, Hill A, Tatem AJ, Wright J (2020). The influence of distance and quality on utilisation of birthing services at health facilities in Eastern Region, Ghana. BMJ Glob Health.

[54] Steinbrook E, Min MC, Kajeechiwa L, Wiladphaingern J, Paw MK, Pimanpanarak MPJ, Hiranloetthanyakit W, Min AM, Tun NW, Gilder ME (2021). Distance matters: barriers to antenatal care and safe childbirth in a migrant population on the Thailand-Myanmar border from 2007 to 2015, a pregnancy cohort study. BMC Pregnancy Childbirth.

[55] Seidu A-A (2021). A multinomial regression analysis of factors associated with antenatal care attendance among women in Papua New Guinea. Public Health Prac.

[56] Muchie KF (2017). Quality of antenatal care services and completion of four or more antenatal care visits in Ethiopia: a finding based on a demographic and health survey. BMC Pregnancy Childbirth.

[57] Sato R, Elewonibi B, Mhande M, Msuya S, Shah I (2019). Canning D: Distance to Facility and Healthcare Utilization in Tanzania: Unbiased and Consistent Estimation using Perturbed Location Data.

