# Supplementary material for: Availability and readiness of healthcare facilities and their effects on antenatal care services uptake in Bangladesh
Source: BMC Health Serv Res. 2024 Apr 5;24:431. doi: 10.1186/s12913-024-10824-4 (PMC10996239; doi:10.1186/s12913-024-10824-4)
Supplement: Supplementary file 1 — Supplementary Material 1. [file 12913_2024_10824_MOESM1_ESM.docx]

**Supplementary Table 1:** Variables included in creating the health facility level factors

| **Name of health facility level factors** | **Number of items** | **Variables considered in generating health facility level factors** |
| --- | --- | --- |
| Basic management and administrative system | 6 | **Supportive management for providers to provide ANC:** availability of healthcare personnel who received training related to ANC during the 24 months preceding the survey, availability of healthcare personnel who received training related to personal supervision during the 6 months preceding the survey, and availability of healthcare personnel who received training related to ANC during the 24 months and personal supervision during the 6 months preceding the survey.  **Infrastructure:** Equipment, knowledge of equipment process time, availability of electricity and alternative. |
| Degree of availability of antenatal healthcare services at the nearest healthcare facility to mothers' homes | 25 | **Trained staffs:** Staff trained for ANC during the past 24 months, staff trained for ANC at any time.  **Equipment:** Blood pressure apparatus, stethoscope, adult weighting scale, fetal stethoscope, measuring tape, examination bed or couch.  **Diagnostic capacity:** Hemoglobin, urine protein, urine glucose, blood grouping and rhesus factor, syphilis.  **Availability of medicine:** Iron tablets, folic acid tablets, combined iron and folic acid, iron or folic acid tablets.  **Infections control items:** Soap, running water, alcohol-based hand disinfect, latex gloves, sharps container, water receptacle. |
| Readiness of the mothers’ homes nearest healthcare facility to provide antenatal healthcare services | 6 | Guidelines on ANC, staff trained for ANC at any time, blood pressure apparatus, hemoglobin testing capacity, urine protein testing capacity, iron or folic acid tablets. |
| Average distance on road communication from women’s resided cluster to the nearest health facility |  | Average distance on road communication from mothers’ resided cluster to the nearest health facility. |
